# Regenerative potential of human muscle stem cells in chronic inflammation

**DOI:** 10.1186/ar3540

**Published:** 2011-12-15

**Authors:** Bouke J Duijnisveld, Anne Bigot, Karel GM Beenakker, Débora M Portilho, Vered Raz, Huub JL van der Heide, Cornelis PJ Visser, Soraya Chaouch, Kamel Mamchaoui, Rudi GJ Westendorp, Vincent Mouly, Gillian S Butler-Browne, Rob GHH Nelissen, Andrea B Maier

**Affiliations:** 1 Department of Orthopaedics, Leiden University Medical Center, PO Box 9600, 2300 RC Leiden, The Netherlands; 2 Institut de Myologie, Inserm U974, CNRS, Groupe hospitalier Pitié-Salpétrière, Paris, France; 3 Department of Gerontology and Geriatrics, Leiden University Medical Center, 2300 RC Leiden, The Netherlands; 4 Department of Human and Clinical Genetics, Leiden University Medical Center, 2333 ZA Leiden, The Netherlands; 5 Department of Orthopaedics, Rijnland Hospital, Simon Smitweg 1, 2353 GA Leiderdorp, The Netherlands; 6 Netherlands Consortium for Healthy Ageing, Leiden University Medical Center, PO Box 9600, 2300 RC Leiden, The Netherlands

## Abstract

**Introduction:**

Chronic inflammation is a profound systemic modification of the cellular microenvironment which could affect survival, repair and maintenance of muscle stem cells. The aim of this study was to define the role of chronic inflammation on the regenerative potential of satellite cells in human muscle.

**Methods:**

As a model for chronic inflammation, 11 patients suffering from rheumatoid arthritis (RA) were included together with 16 patients with osteoarthritis (OA) as controls. The mean age of both groups was 64 years, with more females in the RA group compared to the OA group. During elective knee replacement surgery, a muscle biopsy was taken from the distal *musculus vastus medialis*. Cell populations from four RA and eight OA patients were used for extensive phenotyping because these cell populations showed no spontaneous differentiation and myogenic purity greater than 75% after explantation.

**Results:**

After mononuclear cell explantation, myogenic purity, viability, proliferation index, number of colonies, myogenic colonies, growth speed, maximum number of population doublings and fusion index were not different between RA and OA patients. Furthermore, the expression of proteins involved in replicative and stress-induced premature senescence and apoptosis, including p16, p21, p53, hTERT and cleaved caspase-3, was not different between RA and OA patients. Mean telomere length was shorter in the RA group compared to the OA group.

**Conclusions:**

In the present study we found evidence that chronic inflammation in RA does not affect the *in vitro *regenerative potential of human satellite cells. Identification of mechanisms influencing muscle regeneration by modulation of its microenvironment may, therefore, be more appropriate.

## Introduction

Muscle weakness is a common clinical feature following injury, in neuromuscular diseases and aging leading to disability and increased mortality [1-3]. Understanding cellular mechanisms that regulate loss and gain of muscle mass is, therefore, crucial for treating muscle wasting-associated disorders. The regenerative potential of skeletal muscle is determined by muscle stem cells, which are called satellite cells. These are quiescent mononucleated cells that are sequestered between the basal lamina and the plasma membrane of the myofibers [4]. In response to injury, they become activated, proliferate, differentiate and fuse to existing muscle fibers or fuse together to form new myofibers during regeneration of damaged skeletal muscle [5].

Potential explanations for the decline in muscle mass and strength are multiple factors, including stiffness, immobility, pain, metabolic, hormonal and nutritional status [6,7]. These factors could influence muscle regeneration indicated by the number of satellite cells and their proliferative capacity, which in humans is limited by the mitotic clock [8,9]. Heterochronic parabiosis has been shown to restore the regenerative potential of aged satellite cells which suggest that satellite cell activity can be modulated by the microenvironment and circulating factors [9]. There is growing evidence that chronic inflammation can produce a profound systemic modification of the cellular microenvironment which could affect survival, repair and maintenance of muscle cells [10]. Concentrations of pro-inflammatory cytokines have been shown to increase with advancing age and result in a higher catabolic rate and loss of muscle mass [11,12]. The underlying factors influencing the regenerative potential of satellite cells have not yet been identified.

In the present study, we aimed to define the role of chronic inflammation on the regenerative potential of satellite cells in human muscle. As a model for *in vivo *chronic inflammation, we have used muscle biopsies obtained from patients suffering from rheumatoid arthritis (RA) compared to patients with osteoarthritis (OA), without signs of chronic inflammation. Patients with RA have been shown to have a steeper decline in muscle mass and strength compared to the general population, which might be due to the chronic inflammatory state of these patients [13,14].

## Material and methods

### Subjects

The total study population included 11 patients with RA and 16 patients with OA as controls. After obtaining written informed consent, a muscle biopsy (approximately 420 mg) was taken from the distal *musculus vastus medialis *during elective knee replacement surgery. Pre-operatively, blood samples were taken for analysis of C-reactive protein (CRP), using an immunoturbidimetric method, erythrocyte sedimentation rate (ESR) using the Westergren method and the number of leucocytes using flow cytometry. The study was approved by the medical ethics committee of the Leiden University Medical Center.

### Cell cultures

Myoblast explantation, isolation and cell cultures were performed as previously described [15-18]. Muscle biopsies from the *musculus vastus medialis *were dissected from connective tissue and fat, finely minced and then plated onto non-coated Petri dishes with growth medium consisting of Dulbecco's modified Eagle medium (DMEM, 61965, Invitrogen, Carlsbad, CA, USA), 16% medium 199 (41150, Invitrogen), 20% fetal calf serum (FCS, 10270, lot 41Q4096K, Invitrogen) and 50 ng/ml gentamicin (15750, Invitrogen) supplemented with 5 ng/ml hepatocyte growth factor (PHG0354, Invitrogen). Once mononucleated cells had migrated out from the explants, they were removed from the dish by trypsinization, using 0.05% trypsin-ethylenediaminetetraacetic acid (trypsin-EDTA, 25300, Invitrogen). Mononucleated cells were filtered (40 μm) and plated as a mixed culture in growth medium. At the time of cell isolation, all cell populations were considered to be at one population doubling. All cultures were incubated at 37°C in a humid air atmosphere containing 5% CO_2_. Cell populations were trypsinized when they reached 80% of confluence. Myogenic purity of the populations was determined by immunocytochemistry. To improve myogenic purity, cell populations were magnetically labelled by 15 minutes incubation with 20 μl CD56-micro beats per 10^6 ^cells (130-050-401, Multenyi Biotec, Paris, France). Cell populations were washed with buffer consisting of phosphate buffered saline (PBS, 20012, Invitrogen), 0.5% FCS and 2 mM EDTA and CD56 cell selection was carried out using the MiniMACS (Multenyi Biotec). After determination of the myogenic purity, cell populations were frozen in 90% FCS and 10% dimethyl sulphoxide hybri-max (D2650, Sigma-Aldrich, St. Louis, MO, USA) and preserved at -135°C. Only cell populations with no spontaneous differentiation after trypsinization (less than 10 myotubes with more than 2 nuclei in a 100 mm petri dish) and myogenic purity greater than 75% after CD56 cell selection were used for further analysis. To optimize the comparison of RA and OA patients, all experiments were performed in a highly standardized manner in three batches each containing at least one RA and one OA patient. At the early replicative phase (mean 10.3 (SD 1.7) population doublings (PDs)), myogenic purity, viability, proliferation index, colony formation and growth speed was measured. Protein analysis and telomere length analysis were performed at mean 14.4 (SD 3.1) PDs. Myogenic purity was again measured at the mid-term replicative phase (mean 24.3 (SD 4.2) PDs) and at the end of life span (maximum replicative capacity). The experiments were performed and analyzed blinded for patient diagnosis and age.

### Myogenic purity

Myogenic purity of the cell cultures was determined by immunocytochemistry. Cells were rinsed in PBS, fixed with ethanol (100%) and incubated for one hour with monoclonal mouse D33 (M0760, 1/50, Dako, Trappes, France) as primary antibody specific for desmin, which is only expressed in myogenic cells. Specific antibody binding was revealed in fluorescence by Alexa fluor 488 goat anti mouse antibody (1/750, A11029, Molecular Probes, Eugene, OR, USA) and nuclei were revealed by Hoechst (1/2500, 33258, Sigma-Aldrich) staining. Myogenic purity was calculated as the percentage of desmin positive cells divided by the total number of nuclei.

### Growth characteristics

To measure the proliferation index, cells were cultured for 48 hours in culture medium supplemented with 10 μg/ml bromodeoxyuridine (BrdU, B5002, Sigma-Aldrich). Next, the cells were rinsed in PBS and fixed with ethanol (100%). To render incorporated BrdU accessible to antibody, fixed cells were treated with 2 M HCl for 30 minutes at room temperature and were then neutralized by three 20-minute washes in 50 mM NaCl, 100 mM Tris HCl pH 7.5. Cells were incubated for one hour with a monoclonal antibody directed against BrdU (Bu20a, 1/40, M0744, Dako). Specific antibody binding was revealed in fluorescence by Alexa fluor 488 goat anti-mouse antibody and nuclei were revealed by Hoechst staining. The proliferation index was calculated as the percentage of BrdU positive cells divided by the total number of nuclei. The proliferation index was determined in three experimental triplicates counting at least 500 nuclei in each experiment.

Five hundred myoblasts were seeded on a 100 mm dish (353003, Becton Dickinson, Le Pont de Claix, France) and cultured for 14 days in growth medium to allow the formation of colonies. During this period the medium was not changed. Next, cells were rinsed in PBS, fixed with ethanol (100%) and stained overnight with Giemsa's azur eosin methylene blue solution (109204, Merck, Darmstadt, Germany). Colonies were defined as a cluster of cells containing 16 cells or more. The total number of colonies formed was counted in experimental triplicates. The percentage of myogenic colonies was detected by immunocytochemistry using desmin (M0760, 1/50, Dako).

Growth speed was calculated by the number of PDs per day during the early replicative phase. The number of PDs at each passage was calculated as log(*N/n*)/log 2 where *N *is the number of cells counted at the time of passage and *n *is the number of cells initially plated. During long-term culturing, cells were continuously fed in a standardized manner by seeding 50,000 cells in a 100 mm dish (353003, Becton Dickinson) and serial passaged when they reached 80% confluence until the end of their replicative lifespan. Cultures were considered to be senescent when they failed to divide during three weeks of re-feeding or if the myogenic purity was decreased to less than 10%.

### Fusion index

To induce differentiation of the mononucleated cells into multinucleated myotubes, cells were densely seeded in triplicates on a 12-well dish (353043, Becton Dickinson, 17,000 cells/cm^2^). Once confluent, growth medium was replaced by differentiation medium consisting of DMEM, 10 μg/ml insulin (I550-250 M, Sigma-Aldrich), 100 μg/ml transferrin (T4382-IG, Sigma-Aldrich) and 50 ng/ml gentamicin. After 120 hours, cells were rinsed in PBS and fixed with ethanol (100%). To stain the myotubes, polyclonal mouse MF20 antibody (1/80, Developmental Studies Hybridoma Bank, Iowa City, IA, USA) was used as the primary antibody and Alexa fluor 488 goat anti mouse as the secondary antibody (A11029, 1/750, Molecular Probes). Nuclei were revealed by Hoechst staining. The fusion index was calculated as the percentage of nuclei incorporated into myotubes (> 2 nuclei) to the total number of nuclei. The fusion index was determined in three experimental triplicates counting at least 500 nuclei in each experiment.

### Protein analysis

Cultures were washed in PBS, scraped in 1 ml ice-cold PBS, centrifuged at 800 rpm for one minute and cell pellets were stored at -80°C for further analysis. Cell pellets were resuspended in 150 μL of RIPA buffer (150 mM NaCl, 5 mM EDTA, 50 mM HEPES, 0.5% sodium deoxycholate, 1% NP-40, 0.1% SDS and complete mini-protease inhibitor) and protein concentration was determined with the Pierce BCA kit (Thermo Fisher Scientific, Rockford, IL, USA), using bovine serum albumin as a standard. Sample buffer (4% SDS, 20% glycerol, 0.2 M dithioethreitol, 125 mM Tris-HCl, pH 6.8) was added to aliquots of cell extracts and boiled for five minutes. Twenty μg of protein were loaded on 12% SDS-polyacrylamide gels (SDS-PAGE). Following migration, proteins were transferred to nitrocellulose membranes. The proteins immobilized on the membranes were immediately blocked for one hour at room temperature with a solution containing 5% non-fat dry milk in tris buffered saline containing 0.1% tween 20 (PBT). The blocked membranes were then incubated with a rabbit polyclonal anti-p16 antibody (1/1000, Santa Cruz Biotechnology, Santa Cruz, CA, USA) and mouse monoclonal anti-beta-tubulin antibody (1/1000, clone TUB 2, T4026, Sigma-Aldrich) as the primary antibody and anti-rabbit or anti-mouse peroxidase conjugated antibody (1/40.000, GE Healthcare, Orsay, France) as the secondary antibody. Anti-p16 and anti-tubulin antibodies were visualized using the ECL plus Western Blotting Detection System (GE Healthcare). For p21, p53, cleaved caspase-3 and hTERT detection, PDVF membranes were stained for one minute with 0.1% Ponceau (Sigma-Aldrich). Mouse anti-p21 antibody (CP74, 1/250, kindly provided by A.G. Jochemsen of the Department of Molecular Cell Biology, LUMC), mouse anti-p53 antibody (1/500, Santa Cruz Biotechnology), rabbit anti-human-cleaved caspase-3 (H269518, 1/250, R&D Systems, Abingdon, UK), rabbit anti-hTERT (1531-1, 1/500, Epitomics, Burlingame, CA, USA) and monoclonal mouse anti-muscle actin (1/1000, Santa Cruz Biotechnology) were used as primary antibodies and fluorochrome conjugated goat anti-mouse or goat anti-rabbit as secondary antibodies (1/2000, Licor Biosciences, Lincoln, NE, USA). Quantification of protein expression was performed using ImageJ http://rsb.info.nih.gov/ij/ with data obtained from two experiments. Tubulin or Actin signals were used for normalization.

### Telomere length

Telomere length was measured by flow cytometry using fluorescence *in situ *hybridization with fluorescein-conjugated peptide nucleic acid (PNA) as the probe (K5327, Dako) according to the manufacturer's protocol. In short, cultured cells were trypsinized, mixed with the reference cell line (line 1301; Banca Biologica e Cell Factory, Genoa, Italy), and hybridized with and without Cy3-labeled peptide nucleic acid probe. After labelling the cells with propidium iodide (PI) for DNA content, acquisition was performed using a FacsAria flow cytometer (Becton Dickinson) equipped with Diva software. The probe signal was measured in the FL-1 channel and the propidium iodide signal in the FL-3 channel. Experimental duplicates were performed and results were analyzed according to the manufacturer's protocol.

### Oxidative stress

Twenty-four hours after seeding, cell cultures were exposed to a single acute oxidative stress of 250 μM H_2_O_2 _(H-1009, Sigma-Aldrich) diluted in DMEM with 20% medium 199 and 50 ng/ml gentamicin for 60 minutes at 37°C. Next, cell cultures were rinsed with PBS and cultured in growth medium. Control and stressed cell cultures of three RA and four OA patients followed the same treatment protocol in triplicates with and without the addition of H_2_O_2 _respectively. During the early replicative phase (mean 10.3 (SD 1.7) PDs), myogenic purity, viability, proliferation index, colony formation and growth speed were measured. The percentage of viable cells was determined 24 hours after oxidative stress by trypan blue (T8154, Sigma-Aldrich) exclusion.

### Statistical analysis

Data are presented as mean and standard deviation. Experimental triplicates or duplicates were averaged for statistical analysis. A linear regression model was performed with adjustment for age and gender, myogenic purity, number of PDs and batch. The *P-*value tested the null hypothesis that the diagnosis (RA) does not influence the outcome parameter. All *P-*values below 0.05 were considered statistically significant.

## Results

To investigate the regenerative potential of satellite cells in chronic inflammation, 11 RA patients were included in this study together with 16 OA patients as controls who were all scheduled for elective knee replacement surgery (Table 1). The mean age of both groups was 64 years, with more females in the RA group (91%) compared to the OA group (56%). The mean disease duration in the RA group was 16 years (SD 13). Pre-operative blood samples showed a higher level of inflammatory markers, including a higher CRP, ESR and leucocyte level compared to the OA group. During elective knee replacement surgery, a muscle biopsy (approximately 420 mg) was taken from the distal *musculus vastus medialis*. After mononuclear cell explantation, the mean myogenic purity of the RA group was 34% (SD 20) with 18% spontaneous differentiation in the cell cultures compared to 46% (SD 26) mean myogenic purity in the OA group with 19% of the samples showing spontaneous differentiation. Cell strains without spontaneous differentiation of satellite cells and myogenic purity greater than 75% after CD56 cell selection were considered to be sufficiently pure for further experiments. The resulting experimental group included four RA and eight OA patients. As shown in Table 1 this selected study population had the same distribution in age, gender, disease duration and inflammatory markers as the total study population.

**Table 1:** Characteristics of patients and satellite cell isolation

	Total study population		Selected study population	
	
	RA *N *= 11	OA *N *= 16	RA *N *= 4	OA *N *= 8
Age (years)	64 (12)	64 (9)	65 (9)	64 (9)
Gender (% female)	91	56	75	50
Disease duration (years)	16 (13)	-	15 (13)	-
CRP (mg/L)	12 (11)	2 (3)	10 (7)	2 (3)
ESR (mm)	28 (13)	11 (6)	28 (15)	11 (7)
Leucocytes (*10^9^/L)	10.5 (2.5)	7.0 (1.6)	9.7 (1.4)	7.2 (1.6)
Myogenic purity after explantation (% desmin positive cells)	34 (20)	46 (26)	46 (13)	58 (26)
Spontaneous differentiation* (%)	18	19	0	0
Sufficient biopsy quality# (%)	36	50	100	100

CRP, C-reactive protein; ESR, erythrocyte sedimentation rate; N, number of subjects; OA, osteoarthritis; RA, rheumatoid arthritis. Values are given as mean (SD) if not otherwise stated.

CRP and leukocyte data were available for 14 and 10 patients in the OA and RA group respectively. ESR data were available for 7 patients in OA group and 10 patients in the RA group. *Spontaneous differentiation was defined as 10 or more myotubes with more than 2 nuclei in a 100 mm culture dish. #Sufficient biopsy quality was defined as no spontaneous differentiation and myogenic purity higher than 75% after CD56 cell selection.

### Satellite cell characteristics

**Figure 1:**
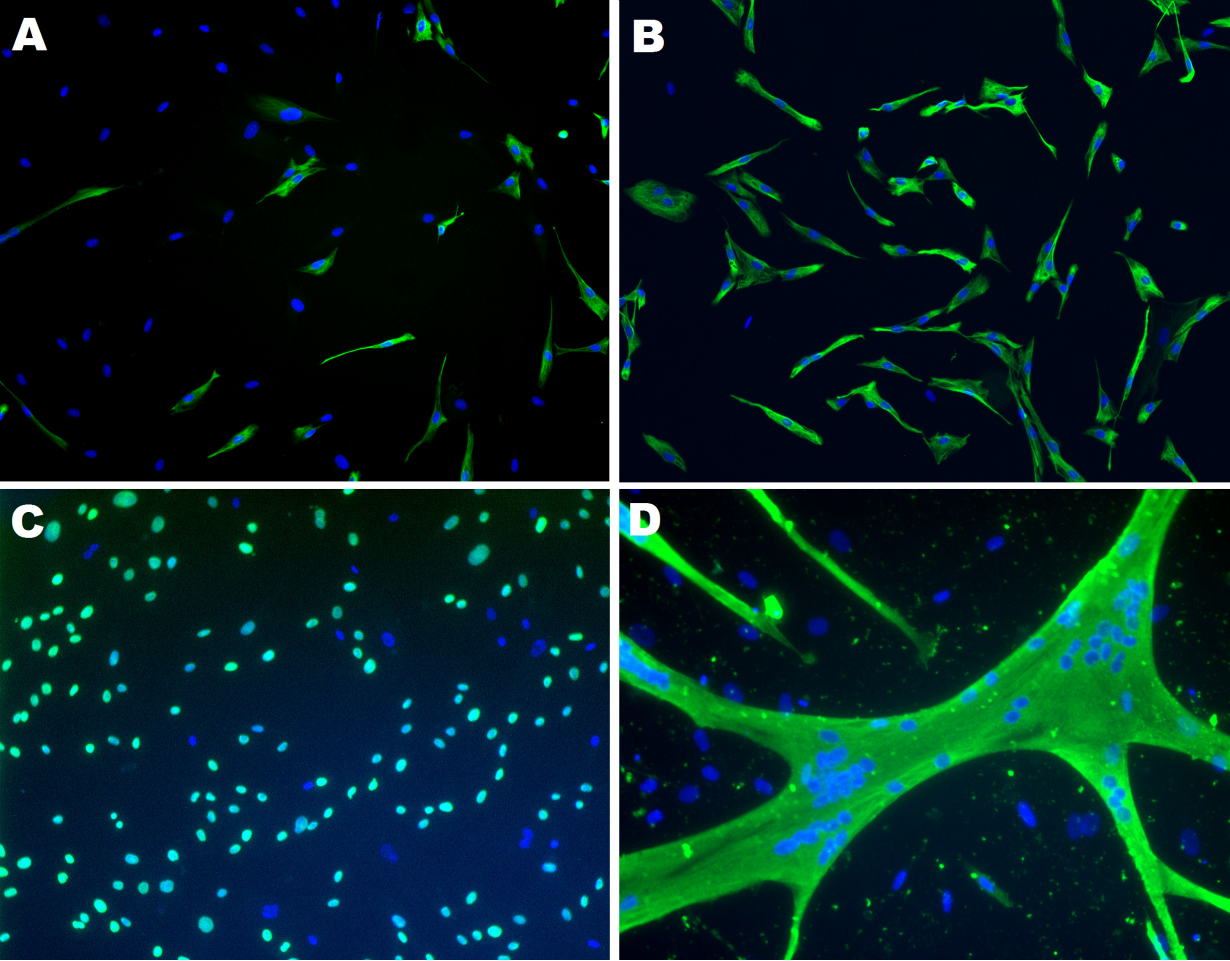
***In vitro *characterization of human satellite cells**. Representative images of *in vitro *characterization of cultured myoblasts using immunofluorescence with an antibody against desmin **(A and B)**, BrdU **(C) **and MF20 **(D)**. Specific antibody labeling was revealed using Alexa fluor 488 green fluorochrome. Nuclei were visualized with Hoechst (blue). Myogenic purity was determined after explantation (A) and increased after CD56 selection (B). To determine the proliferation index, cells were cultured for 48 hours in culture medium supplemented with 10 μg/ml BrdU (C). To determine the fusion index, cells were cultured in differentiation medium for 120 hours (D). Original magnification 100 × (A, B and C) and 100 × (D).

**Figure 2:**
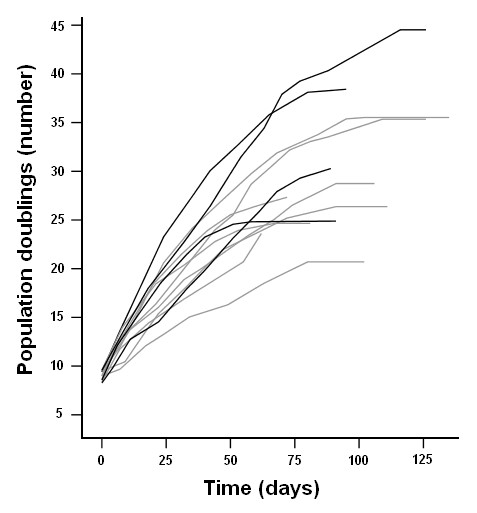
**Growth curve of human satellite cells**. Growth curve from thawing (Day 0) until maximum replicative capacity of human satellite cell strains from patients suffering from rheumatoid arthritis (*N *= 4, black lines) or osteoarthritis (*N *= 8, gray lines).

To investigate the influence of chronic inflammation on human satellite cell regenerative potential, multiple satellite cell characteristics were measured (Table 2, Figure 1). During the early replicative phase (mean 10.3 (SD 1.7) PDs) myogenic purity, viability, proliferation index, mean number of colonies, myogenic colonies, growth speed and fusion index were not different between RA and OA patients as was the myogenic purity at mid-term and at replicative senescence. As depicted in Figure 2, the growth curves showed no difference between the RA group with a mean maximum number of 33.4 (SD 9.7) PDs and the OA group with mean maximum of 27.4 (SD 5.5) PDs. After adjustment for age, gender, myogenic purity, number of PDs and batch, none of these parameters were statistically significantly different between RA and OA patients (data not shown).

**Table 2:** Characteristics of satellite cell strains obtained from rheumatoid arthritis patients and osteoarthritis patients

Characteristic	RA *N *= 4	OA *N *= 8	β	95% CI	*P*-value
Myogenicity (% desmin positive cells)					
After explantation	46 (13)	58 (26)	-0.35	-51 to 19	0.32
After CD56 selection	87 (5)	95 (3)	-0.72	-13 to -2	0.02
After thawing at early replicative phase*	60 (20)	47 (32)	-0.57	-209 to 142	0.62
At mid-term replicative phase#	56 (37)	37 (33)	-0.02	-49 to 46	0.95
At maximum replicative capacity	47 (43)	38 (30)	-0.07	-86 to 77	0.90
After oxidative stress†	21 (12)	16 (10)	0.21	-341 to 349	0.91
Viability (% alive cells)					
At early replicative phase*	91 (4)	94 (2)	-0.39	-8 to 4	0.33
After oxidative stress†	33 (24)	34 (24)	0.48	-114 to 153	0.31
Growth characteristics*					
Proliferation index‡ (% BrdU positive cells)	86 (2)	85 (6)	-0.05	-10 to 9	0.89
Colony formation (number)	78 (44)	85 (34)	-0.12	-83 to 66	0.78
Myogenic colonies (%)	30 (16)	27 (31)	-0.40	-41 to 7	0.14
Growth speed (PD/day)	0.39 (0.10)	0.32 (0.08)	0.310.28	-0.07 to 0.18-5.0 to 13.9	0.290.28
Maximum replicative capacity (PD)	33.4 (9.7)	27.4 (5.5)	-0.29	-385 to 359	0.73
Proliferation index after oxidative stress†‡ (% BrdU positive cells)	15 (15)	34 (24)			
Fusion index at early replicative phase*§ (% of fused myonuclei)	27 (12)	25 (13)	-0.01	-26 to 26	0.99
Normalized protein expression*					
p16	103 (15)	140 (63)	-0.62	-85 to 7	0.08
p21	27 (4)	27 (5)	-0.28	-13 to 8	0.60
p53	67 (12)	62 (8)	-0.18	-18 to 11	0.58
hTERT	32 (6)	39 (14)	-0.26	-23 to 11	0.39
Relative telomere length* (% of ref. cell line 1301)	22 (5)	23 (10)	-0.46	-16 to -0.4	0.04

N, number of subjects; OA, osteoarthritis; RA, rheumatoid arthritis. Beta (β). 95% confidence interval (CI) and *P*-value are shown for linear regression model adjusted for age and gender, myogenic purity, number of PDs, and batch. Values are given as mean (SD) if not otherwise stated. *Early replicative phase: mean 10.3 (SD 1.7) PDs for myogenic purity, viability, growth characteristics and fusion index; mean 14.4 (SD 3.1) PDs for normalized protein expression and relative telomere length. #Mid-term replicative phase: mean 24.3 (SD 4.2) PDs. †Myogenic purity, viability and proliferation index was measured at early replicative phase, 24 hours after oxidative stress (one hour of 250 μM H_2_O_2 _at 37°C) in four OA and in three RA patients. ‡Proliferation index was determined as the percentage of bromodeoxyuridine (BrdU) positive cells after 48 hours of culture with BrdU. §Fusion index was determined as the percentage of fused nuclei (> 2 nuclei per myofiber) in MF20 positive cells.

#### Senescence-associated proteins apoptosis and telomere length

**Figure 3:**
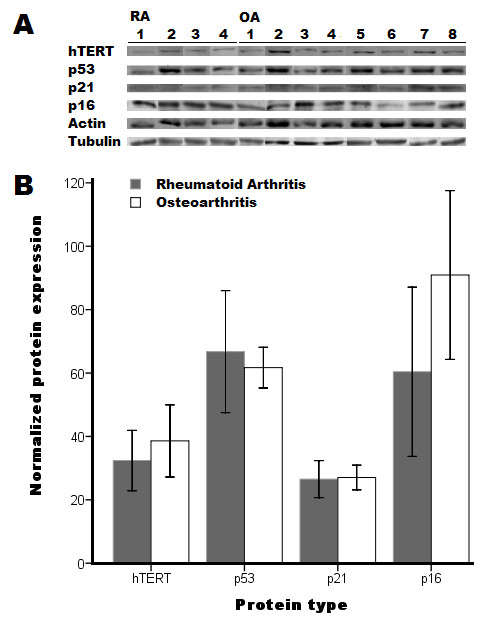
**Protein expression of human satellite cells**. **A: **Representative image of hTERT, p53, p21 and p16 expression in human satellite cells from patients suffering from rheumatoid arthritis (*N *= 4) or osteoarthritis (*N *= 8). Actin and tubulin expression was used for normalization. **B: **Normalized average protein expression and 95% confidence interval of hTERT, p53, p21 and p16.

**Figure 4:**
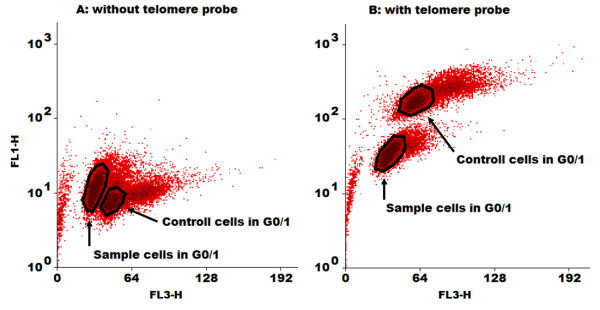
**Telomere length of human satellite cells**. Telomere length was measured by flow cytometry using fluorescence *in situ *hybridization without **(A) **or with the fluorescein-conjugated PNA probe **(B)**. Propidium iodide (PI) was used for labeling DNA content. The telomere probe signal was measured in the FL-1 channel and the PI signal in the FL-3 channel. G0/1 phase sample and control cells were gated according to their PI specific signal on a dot blot (FL1-H vs. FL3-H). Relative telomere length (RTL) was calculated by: RTL = (mean FL1 sample cells with probe - mean FL1 sample cells without probe)/(mean FL1 control cells with probe - mean FL1 control cells without probe) * 2 * 100%. Representative images are shown of the one of the RA patients. For each sample analyzed 20,000 counts were acquired.

To determine whether senescence is induced in satellite cells, the expression of hTERT, p53, p21 and p16 proteins was determined at the early replicative phase using a Western blot analysis. Averages of normalized protein expression did not significantly differ between RA and OA patients (Table 2, Figure 3). In addition, none of the samples were positive for cleaved caspase-3 expression. Furthermore, the mean relative telomere length was not different between RA and OA patients (Table 2, Figure 4). After adjustment for age, gender, myogenic purity and batch, the mean relative telomere length was lower in the RA group compared to the OA group (β = -0.46, 95% CI -16 to -0.4%, *P *= 0.04). The other measurements were not different. The maximum replicative capacity of the satellite cell strains was not significantly associated with telomere length (β = 0.60, 95% CI -1.3 to 2.3%, *P *= 0.48).

#### Oxidative stress

To determine whether satellite cells from RA patients were more susceptible to oxidative stress, we administered a single dose of 250 μM H_2_O_2 _for 60 minutes at 37°C to the cell cultures. Twenty-four hours after oxidative stress, the myogenic purity, viability and proliferation index were decreased in both the RA and OA group. None of the cell strains were able to reach confluence after three weeks or to form colonies after oxidative stress. The satellite cells from the RA group were as susceptible to oxidative stress as the OA group as shown in Table 2, also after adjustment for age, gender, batch and myogenic purity (data not shown).

## Discussion

The regenerative potential of human muscle stem cells is thought to be influenced by their microenvironment, including cytokines. To determine the potential deleterious effect of inflammation on the regenerative capacity of skeletal muscle, we compared muscle biopsies obtained from patients with chronic inflammation (RA) to control patients (OA). We found no differences in myogenic purity, viability, growth speed, differentiation and maximum proliferative capacity. These results were confirmed by no difference in major pathways involved in muscle regeneration, including the expression of proteins involved in replicative and stress induced premature senescence and apoptosis. Telomere length was shorter in RA patients compared to controls.

Mechanisms involved in the regenerative potential of muscle include the ability to restore the quiescent satellite cell pool which has been shown to decrease during aging by a decrease in the number of satellite cells present in muscle biopsies [9,14]. Recently, we have shown that chronic inflammation does not influence the number of satellite cells in human muscle (data submitted for publication). In the present study, we provide new evidence that the *in vitro *regenerative potential of human satellite cells seems not to be affected by chronic inflammation.

Mechanisms affecting the regenerative potential of muscle include the number of satellite cells which are able to proliferate, their growth speed and their maximum proliferative capacity which have been shown to be affected by age [8]. In the present study we demonstrate *in vitro*, that chronic inflammation *in vivo *does neither affect any of the growth characteristics nor the differentiation potential of human satellite cells. Regenerative potential of human satellite cells is limited by the progressive erosion of their telomeres after each cell division leading to critically short telomere length and the activation of replicative senescence through a p53 and p21 dependent pathway [19]. The erosion of telomeres can be prevented by the catalytic subunit of the telomerase (hTERT) leading to extension of replicative life [20]. The mean relative telomere length in RA patients was significantly lower after correction for age, gender, myogenic purity, batch and number of population doublings, which could suggest an increased turnover of these cells. However, this difference was minimal and the expression of p53, p21 and hTERT proteins were not different between RA and OA patients. Therefore, we conclude that chronic inflammation does not significantly increase nuclear turnover which is in agreement with our findings regarding the growth characteristics of satellite cells cultures. Stress induced premature senescence has been shown to be induced by p16 in aging and myotonic dystrophy. Here we demonstrate that p16 is not increased in chronic inflammation [17,21]. Apoptosis and programmed cell death was not detected during the amplification of either chronic inflammation (RA) or control cells (OA) using anti-cleaved caspase 3 [22].

An unexpected finding was the low mean myogenic purity after explantation in both groups, as myogenic purity was shown to be high in both young and old age, which could have been due to the different biopsy material and procedures used in the different studies [8]. To optimize myogenic purity, we performed a CD56 magnetic selection procedure and only cell populations with a mean myogenic purity higher than 75% were included in our study. Some cell cultures showed spontaneous differentiation and were, therefore, excluded. Because of low myogenic purity and increased spontaneous differentiation, we were able to obtain fewer biopsies in the RA group than in the OA group for *in vitro *experiments. Although the difference in muscle biopsy quality was not significant, this could indicate that chronic inflammation does influence satellite cell behavior and ultimately the regenerative potential of the muscle. Non-myogenic stem cells have, however, been shown to contribute to muscle regeneration [23]. After thawing, we also observed a decrease in mean myogenic purity in both groups. Freezing could have led to stress by cold shock, ice formation, activation of apoptosis mechanisms and alteration of certain signalling pathways. Fetal and newborn muscle cells have been shown to conserve their high myogenic purity after long-term cryopreservation; however, adult cells are more difficult to explant and have a lower myogenic purity [24]. In the present study, we did not observe a correlation between myoblast telomere length and replicative capacity. Furthermore, we did not observe a correlation between telomere length and other markers of replicative capacity like the expression of p21, p53 or hTERT. A potential explanation involves the induction of pathways involved in stress induced senescence, being telomere length independent, which cannot be excluded during *in vitro *replicative senescence [25]. Serial culturing represents a major difference from the *in vivo *environment, including changes in their physical environment, the cell to cell interaction and different nutrition supply, which could induce oxidative stress, DNA damage and, finally, stress induced premature senescence [26,27].

As a model for chronic inflammation, the study included patients suffering from RA who indeed showed a higher level of inflammation compared to OA patients as indicated by CRP, ESR and number of leucocytes. Furthermore, the two groups showed the same age distribution. Although OA is associated with local inflammation [28], we did not use a healthy control group because such a control group is physically more active, which would then possibly confound the results [29]. Furthermore, healthy patients without an underlying chronic condition are not available for the same muscle biopsy area, technique and biopsy size. The strength of this study is the rigorous standardization in terms of patient selection, muscle biopsy location, cell culture experiments in batches with both RA and OA patients and the extensive phenotyping of the satellite cell populations. The limitations of this study are that the influence of chronic inflammation on the *in vitro *regenerative potential of human satellite cells may not be found due to the selection of muscle biopsies with good quality. Secondly, the *in vitro *experiments might not reflect the *in vivo *situation and finally the small number of included patients made it impossible to differentiate within and between the groups on the presence of biological treatment (for example, anti TNF). In addition, it should be noted that there was a certain selection since the myogenic purity was lower in the RA group and many cells were lost to spontaneous differentiation which could be due to an increased expression of p16. However, including more patients was not possible due to the extensive phenotyping of each satellite cell strain. Furthermore, the RA group included more females (75%) compared to the OA group (50%). Myogenic purity and gender were adjusted for in the linear regression model and had no effect on satellite cell regenerative potential *in vitro*.

Muscle weakness is an increasing clinical problem and occurs upon injury, neuromuscular diseases and aging, leading to disability and increased mortality [1-3,30,31]. Stem cell therapy is one possible strategy to regenerate muscle. Although many studies with different types of stem cells have been conducted, the efficacy of cell therapy is still limited by homing and a reduced ability to resist the environment of the damaged muscle [32]. Conboy *et al*. showed that heterochronic parabiosis could restore the regenerative capacity of aged satellite cells [33]. The circulating factors presented by a shared circulatory system could be the possible explanation for the observed rejuvenation. Therefore, the microenvironment of muscle in which pro-inflammatory cytokines have been shown to result in a higher catabolic rate and loss of muscle mass might be crucial for the regenerative potential [11,12]. One of the hallmarks of inflammation is the release of interleukins and tumour necrosis factor-α (TNFα) attracting macrophages which are critical in the repair of skeletal muscle by the release of cytokines and growth factors and by phagocytosis. However, in their attempt to enhance tissue repair they may also destroy surrounding healthy muscle fibers [34]. Furthermore, TNF-α induces activation of transcription factor NF-κB, which results in activation of the ubiquitin-proteasome pathway and breakdown of MyoD and myogenin, which are essential regulators for satellite cell differentiation [35] as well as contractile proteins essential in maintaining muscle mass. Inflammatory factors could be the cause of lower muscle strength of patients with RA compared to OA [14].

*In vitro*, our results demonstrate no difference in potential regenerative capacity between satellite cells from patients with chronic inflammation and control patients. This could be due to the fact that the satellite cells of both patient groups were put in culture and, therefore, both experienced an optimized microenvironment for proliferation and differentiation. Consequently, our results show that there is no difference in satellite cell behavior *in vitro*, indicating that circulating inflammatory factors *in vivo *could be the cause of the decrease in muscle strength. Further evidence of the *in vivo *influence of inflammatory cytokines has been shown by anti-inflammatory treatment, which has been shown to improve muscle regeneration after injection with mesoangioblasts [36].

## Conclusions

In conclusion, in the present study we found evidence that chronic inflammation in RA does not affect the *in vitro *regenerative potential of human satellite cells. This result underscores the *in vivo *influence of inflammatory factors on muscle regeneration. Identification of mechanisms influencing muscle regeneration by modulation of its microenvironment may improve strategies to regenerate human muscle, in particular in conditions such as muscle injury, neuromuscular disorders and aging.

## Key messages

RA does not affect *in vitro *regenerative potential of human satellite cells compared to controls.

RA does not affect senescence associated protein expression in satellite cells compared to controls.

Telomere length in satellite cells from RA patients is shorter compared to controls.

## Abbreviations

BrdU: bromodeoxyuridine; CI: confidence interval; CRP: C-reactive protein; DMEM: Dulbecco's modified Eagle medium; ESR: Erythrocyte sedimentation rate; FCS: fetal calf serum; N: number of subjects; OA: osteoarthritis; PBS: phosphate buffered saline; PDs: population doublings; PI: propidium iodide; PNA: peptide nucleic acid; RA: rheumatoid arthritis; SD: standard deviation; TNFα: tumour necrosis factor-α.

## Competing interests

The authors declare that they have no competing interests.

## Authors' contributions

BJD, GS B-B, RGJW, RGHHN and ABM developed the study concept and design. BJD, ABM, KGMB, HJLvdH, CPJV and RGHHN were responsible for patient inclusion and muscle biopsy handling. KM, SC and AB were responsible for cell culture MACS, immunocytochemistry before and after purification, tests for mycoplasm, and amplification, freezing and storage of cell samples. BJD, AB and DMP acquired the data. BJD, AB, GS B-B, VM, DMP, VR, RGHHN and ABM analyzed and interpreted the data. BJD and ABM drafted the manuscript. AB, GS B-B, VM, DMP, VR, KGMB, HJLvdH, CPJV, RGJW, RGHHN and ABM provided critical revision of the manuscript for important intellectual content. BJD and ABM carried out statistical analysis. GS B-B, RGJW, RGHHN and ABM supervised the study. All authors approved the final version of the manuscript.
